# When the Going Gets Tough: A Review of Total Hip Arthroplasty in Patients with Ipsilateral Above- and Below-Knee Amputation

**DOI:** 10.3390/medicina60091551

**Published:** 2024-09-22

**Authors:** Alberto Di Martino, Enrico Capozzi, Matteo Brunello, Claudio D′Agostino, Laura Ramponi, Alessandro Panciera, Federico Ruta, Cesare Faldini

**Affiliations:** 1 Orthopedic and Traumatology Clinic, IRCCS Rizzoli Orthopedic Institute, Via G.C. Pupilli 1, 40136 Bologna, Italy; enrico.capozzi@ior.it (E.C.); matteo.brunello@ior.it (M.B.); claudio.dagostino@ior.it (C.D.); laura.ramponi@ior.it (L.R.); alessandro.panciera@ior.it (A.P.); federico.ruta@ior.it (F.R.); cesare.faldini@ior.it (C.F.); 2 Department of Biomedical and Neuromotor Science-DIBINEM, University of Bologna, 40136 Bologna, Italy

**Keywords:** amputated, below-knee-amputated, above-knee-amputated

## Abstract

Life expectancy and overall function of amputated patients have improved significantly over the last few decades; for this reason, amputees are more exposed to primary or secondary degenerative disease of the hip, requiring total hip arthroplasty (THA) surgery. However, during training, not all the surgeons acquire adequate skills to manage these patients, and only a few studies and case reports describe technical pearls and outcomes of THA in patients with ipsilateral lower limb amputation, either above or below the knee. The objective of this narrative review is to present current evidence and surgical tips for performing THA in ipsilateral amputated patients, with a focus on the differences between patients with above- (AKA) and below-knee amputation (BKA). We reviewed manuscripts in major scientific databases, cross-referencing to retrieve adjunctive manuscripts, and summarized all relevant cases. We found 17 manuscripts, spanning 70 years of literature, collecting a total of 39 patients who underwent THA on an ipsilateral amputated limb: 13 AKA, 23 BKA, and 3 through-knee-amputation (TKA). The cohort primarily consists of patients with post-traumatic hip arthritis, often associated with sequelae such as fractures to other bones, soft tissue compromise and heterotopic calcifications. Managing with amputated patients requires careful planning, which includes the study of the residual bone, muscle anatomy, and the level of femoral amputation, as these factors present significant surgical challenges, especially in patients without a knee joint. In dealing with the post-traumatic and multi-comorbidity patients, rehabilitation goals should be considered prior to surgery and should drive the surgical strategy. We found that BKA patients typically have high functional demands, necessitating precise positioning of the components and an aggressive post-operative physiotherapy regimen to avoid unsatisfactory outcomes. AKA patients, on the other hand, often present with altered anatomy, and typically require more surgical instruments and expertise to achieve intraoperative dislocation of the hip joint.

## 1. Introduction

Total hip arthroplasty (THA) is a safe and effective procedure aiming at relieving pain and improving function in patients with end stage osteoarthritis of the hip joint, medial femoral neck fracture, or avascular necrosis of the femoral head [1]. The procedure and implants can be adapted to several types of patients, characterized by different comorbidities, disabilities, or accidents, which can lead to extremely severe complications. Studies have shown an increased risk of developing hip osteoarthritis at the intact limb of an amputated patient [2,3], while others have reported an increased rate of OA in the amputated limb [4]. THA in patients with amputation of the same lower limb, either above or below the knee, requires specific competencies to optimize patients’ recovery and promote ambulation by wearing a prosthesis.

The trend to perform lower limb amputations (LLA) is decreasing; however, they are still commonly performed worldwide. In 2011, approximately 150,000 patients per year [5] underwent a LLA in the United States, and this rate decreased by 45% after 10 years [6]. When European data were analyzed, it was found that in the UK, 11,500 major lower limb amputations were performed each year [7], with major amputation rates that fell by 20% in a ten-year timeframe [8] (2003–2013). In Germany, 62,016 LLA were registered in 2019, and this rate decreased by 7.3% in five years [9].

The most common causes leading to LLA are diabetes mellitus, peripheral vascular disease, neuropathy, and trauma. The few available reports describe the outcomes of THAs, mainly in this latter patients’ population [4], including veterans and those undergoing high energy traumas; these types of amputated patients often sustain fractures at other skeletal segments, including pelvis, acetabulum, and other bones of the lower limb, which can compromise the overall limb alignment and inevitably increase the risk of a disfunction in the hip joint. Amputees may also be at risk for osteoporotic fractures developing even at younger ages compared to non-amputees because of low focal BMD [10] arising from the weight avoidance on the residual limb, and for the increased risk of falls observed in these patients [11,12]. People who underwent a unilateral transtibial amputation (TTA) are 88% more likely to experience osteoporosis at the amputated limb compared to the general population [2]. Moreover, the altered load in the lower limb in amputees is associated to a large decrease in BMD both at the hip and at the stump compared with the intact side, with patients with above-knee amputation (AKA) showing greater bone atrophy than those with below-knee amputation (BKA); this factor increases the risk of femoral neck fractures at the side of the amputated limb [10].

Patients with AKA and BKA require a different approach in terms of surgical performance, gait recovery, and overall post-operative rehabilitation. At present, only case reports and few retrospective studies deal with THA performance and outcomes in patients with ipsilateral limb amputation. Therefore, the aim of the current review is to summarize current knowledge on THA performance on the same side in AKA and BKA patients, describing the main surgical-related issues to optimize surgery, and outlining post-operative care and functional outcomes.

### 1.1. Biomechanics of the Lower Amputated Limb

Gait analysis in the amputated patient at the lower limb is characterized by different motor patterns related to the level of amputation [13]; those mostly affect the walking activity more than the stance pose, with a significant impact on the patient’s more complex motor tasks, including gait initiation and termination, and crossing of obstacles. The gait after unilateral BKA requires less energy, and it closely resembles the gait of healthy individuals when compared to the gait of patients with AKA [14,15].

AKA patients are more prone to developing complications in the thigh, including flexion and abduction contractures at the hip joint, atrophy of the muscles on the amputation stump, and skin damage [16]. After amputation, there is a reduction in muscle mass caused by myodesis techniques, leading to a secondary reduction of muscle torque with atrophy of thigh stump muscles. Specifically, the role of muscles abducting the lower limb at the hip joint is to create tension to the fascia lata during the stance phase, and the muscle torque of the antagonistic muscles is generated mainly by the adductor magnus muscle during the same phase of gait. The balance is therefore strictly related to the level of amputation: the more distal is the level at the thigh, the more effective is the action of the adductor muscles in antagonizing the glutes medium. The loss of the distal third of the femur with compromise of the insertion of the adductor magnus muscle leads to a loss of 70% of effective muscle torque, which determines itself a contracture in flexion and abduction [17]. Gottschalka et al. [17], in an early study in which they examined the biomechanics of the TTA patient, indicated that the weakness of the muscles adducting the lower limb at the hip joint and the excessive strengthening of the abducting muscles can determine hip joint distortion, which alters the gait cycle. On the other hand, any modification improving the strength of the adductors results in improvement of gait symmetry [18].

Hip OA in the amputated limb occurs in approximately 55% of older patients with trans-femoral amputation [4]. A higher prevalence of OA in traumatic amputated patients with ipsilateral AKA and BKA is observed compared to the contralateral side, with a threefold increased risk of OA in AKA compared to BKA. It is widely agreed that habitually increased joint loading is a primary contributor to OA development at the intact limb in patients with AKA [2,3,19]; however, studies diverge on the occurrence of increased loadings at the amputated limb [20]. A recent study by Galen Roda et al. [21] demonstrated how altered loads in ipsilateral AKA determine a variation in the shape of the proximal femur, including loss of sphericity of the femoral head, which can likely contribute to the development or progression of OA.

### 1.2. Surgical Relate Issues

There is no gold standard in terms of surgical approach and implant type for performing THA in amputated patients, because of the low level of evidence on this topic, and because reports encompass different techniques along with specific tips and tricks.

THA surgery in lower limb amputees has been performed by several approaches according to the surgeon’s preference, including posterior [22,23], postero-lateral [24,25], direct lateral [26,27], and antero-lateral [28,29]; this is specifically due to the heterogenous court of studied patients. There is not a clear preference regarding the surgical approach; however, AKA patients may be more suitable to posterolateral approach performance, while BKA can be managed by several approaches, including those with the patient supine, namely direct lateral, anterolateral, and even direct anterior.

More than in conventional THA, pre-operative planning is extremely important, and it should take into consideration some critical aspects. First, hip radiographs [30] should include the complete stump to assess the remaining femoral length and the width of the medullary canal, because some changes in bone mineral density and distal bony geometry at the stump due to prolonged wear have been reported [10]. Secondly, accurate evaluation of the skin condition and local trophism at the stump is required before surgery to limit the risk of infection of THA implants. To reduce the infection risk, apart from the standard pre-operative antibiotic therapy, some authors recommend draping the stump with a plastic adhesive during surgery to isolate its distal part, which is at risk of contamination because of its usual inclusion in the socket of the external prothesis, making it a presumably contaminated zone [31]. In addition, further care should be taken to the skin of the residual limb during closure of the operative wound to avoid troublesome scars that could facilitate infections and conflicts with the prosthesis. Finally, a proper bandage of the stump should always be worn during rehabilitation, especially when using the external prosthesis, to prevent soft tissue infections.

Since amputations are often post-traumatic, malunion of other fractures and presence of heterotopic ossifications are frequently encountered. Soft tissue release, intraoperative tenotomies, or even capsular releases performed for contracture of the hip flexors and abductors are often required. Moreover, when severe soft tissue contractures or heterotopic ossifications resist against the dislocation of the femoral head, an extensive removal of the ossifications, or even an osteotomy of the greater trochanter may be needed [25].

**Figure 1:**
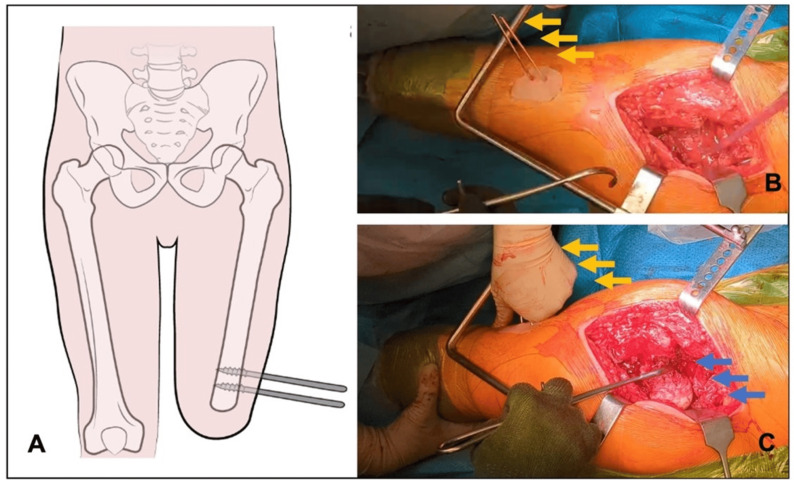
(**A**) In case of total hip arthroplasty in above-knee-amputated patients, the absence of the knee joint may compromise the ability of the surgeon to dislocate the hip. The insertion of two Steinman pins or Shanz screws (**A**) at the distal part of the femur allow to control the intraoperative position of the femur (**A**). Pins (orange arrows) are usually inserted perpendicular to the femur in line with the incision, avoiding the distal part of the stump. (**B**) These pins are used as a lever to intraoperatively control the hip joint, allowing complex movements like femoral head (blue arrows) dislocation through 90° internal rotation of the femur (**C**).

The main issue with AKA is the management of the position of the hip and femur in the absence of the knee; in fact, the presence of the joint hinge allows the assistant surgeon to lever the tight to achieve correct and precise intraoperative position of the femur. In AKA patients, the absence of knee and the tibia determine some serious challenges when it comes to dislocating the joint, controlling the stump, and placing the femoral implant with the right angle of anteversion and alignment. Some surgeons reported the use of a traction pin or a bone clamp in the sub-trochanteric area to have a satisfactory rotation and traction control on the stump [27,28,31]. Even though the positioning of the traction pin is left to the surgeon’s preference, surgeons can usually place a couple of Steinmann pins at the lateral aspect of the distal femur, avoiding conflict with the implant (Figure 1), to improve the leverage available for rotational control [28], carefully leaving intact any osteopenic areas and previous scar tissue. Other surgeons prefer to place it at the greater trochanter level, in part because of femur stump shortness [22], or to reduce the difficulty of incision exposure and risk of infection [23,32]. One other device which is often used in this case is a bone clamp, usually placed more proximally to improve rotational control: some authors place it at the peri-trochanteric region [24] or at the subtrochanteric area [33,34], while others prefer to avoid the trochanteric region because it is considered more fragile; in this case, the clamp is placed at the insertion of the gluteus maximus on the femoral shaft to apply a powerful manipulation of the limb with a minimal risk of bone injury [31].

In BKA patients, the principal difficulty of the surgical procedure is correctly positioning the femoral component [25]; in fact, the stem of the implant should be inserted with a correct alignment, avoiding the risk of retroversion. This is aimed to avoid dislocations and to restore a proper offset, optimizing the function of the hip abductors [35], which is important in BKAs because these are more often active [36], usually gain better mobility [36,37], and generally have higher quality of life [36].

### 1.3. Clinical Studies

#### 1.3.1. Method

The literature review began with a search of the electronic databases PubMed and Google Scholar using various forms and combinations of the key words: total hip arthroplasty (THA), ipsilateral amputation, above-knee-amputated, and below-knee-amputated. The literature search located around 250 publications indexed during the largest time span possible (1967–March 2024), including mostly case reports and fewer small case series and reviews. When appropriate, reference citations from publications identified in the literature search were reviewed. Publications highlighted in this article were extracted based on relevance to the matter, excluding all the studies that describe the THA procedure on the controlater amputated limb, or in patients with both limbs amputated. This was necessary due to our intention to focus on surgical techniques, and highlight the rehabilitation challenges and the possibility of achieving satisfactory ambulation in this review.

#### 1.3.2. Below-Knee Amputees

There are few case reports and patients’ cohorts focusing on THA in BKA patients (Table 1).

One of the earliest reports was described by Prickett et al. in 1972 [38], featuring a 59-year-old woman with a transcervical fracture of the left femur who had undergone BKA 17 years before. A cemented implant was used, allowing for the patient to ambulate on the third post-operative day using a below-knee prosthesis and a walker. During the post-operative rehabilitation, the patient experienced a symptomatic limb length discrepancy and valgus deformity of the left knee. Radiographs revealed a 1.2 cm lengthening of the femur and retroversion of the femoral component. The authors attributed the observed biomechanical changes to the new rotational position of the femoral component that led to adduction of the left femur and valgus positioning of the left knee when weight bearing on the prosthesis.

Salai et al. [29], described a series of five relatively young patients with displaced sub-capital femoral head fractures and previous BKA. THA was performed using a cementless implant using an antero-lateral approach, yielding “excellent” to “good” results based on adjusted Harris Hip Score at an average 5.5-year follow-up. Notably, ambulation without assistance was achieved in two patients, while three patients required a cane. Three out of five patients underwent attempted fracture reduction and fixation before arthroplasty due to their relatively young age, which subsequently failed, requiring THA. Authors suggested that the failure of the internal fixation could be attributed to the altered biomechanics at the fracture site in limbs with BKA, in which vertical excursion is increased and lateral shifting of the center of gravity was observed, determining greater shearing forces at the fracture site, promoting non-union.

BKA may require a longer time to walk independently after THA when compared to intact patients. In the study by Nejat et al. [39], the authors observed how BKAs with THA required an average of 5.5 days for ambulation with a walker compared to non-amputated patients that required an average of 1.2 days to walk after a THA. Of the two reported patients, the first had a left Symes amputation due to congenital dysgenesis of the femur and went through a total left THA, which required three revision surgeries in 20 years. On post-operative day five, the patient was discharged home with crutches, and at the last follow-up, she did not require ambulatory aids. The second patient suffered from osteomyelitis that led to BKA; she required THA because of post-traumatic arthritis after an acetabular fracture that left her bound to a wheelchair. Three months after surgery, she ambulated well with a walker and the prosthesis. Authors suggested that 6 weeks of post-operative intensive physical therapy was crucial for amputees with THAs to prevent dislocation, to which amputees are at risk when removing their prostheses.

Another 84-year-old patient with previous BKA for vascular disease with cardiac comorbidity, developed severe end stage osteoarthritis at the right hip [40]. Ten days before surgery, the patient was trained in muscle strengthening exercises with stretching of the tight flexors of the hip and strengthening of the weak gluteal and hamstring muscles. He then underwent THA surgery via a posterior approach with no major complications. At the 3-month follow-up, a good improvement in the ROM of the hip joint was observed, and the authors emphasized the role of stretching of the hip flexors and knee extensors in the post-operative rehabilitation of BKA patients with an ipsilateral THA.

A 57-year-old female with a history of BKA at the age of 3 underwent ipsilateral cemented THA after a femoral neck fracture [26]. They demonstrated satisfactory outcomes with a notable range of motion, allowing for ambulation with only one cane. The authors refused to use Steinman pins for hip dislocation to avoid infections and risk of fracture at the osteoporotic bone.

#### 1.3.3. Above-Knee Amputees

There are only a few case series reported so far on the results of THA in ipsilateral AKA patients (Table 2).

Amanatullah et al. [41] published a case series in 2015 with 18 THA in amputee patients; fourteen out of these 18 (77.8%) were ipsilateral to a BKA, 2 (11.1%) were ipsilateral to a through the knee amputation, and 2 (11.1%) were ipsilateral to an AKA. A total of 16 were approached by an anterior route, and the other 2 by a posterolateral approach. A total of 10 implants (55.6%) were cemented, while 8 (44.4%) were uncemented. Average pre-operative HHS was 25.4 ± 21.7 (range, 5 to 57), increasing post-operatively to HHS 78.6 ± 17.1 (range, 53 to 100). Four patients (22.2%) had major post-operative complications. The average time of progression to THA after a contralateral lower extremity amputation was 12.2 years, more than double the time of progression (average, 5.4 years) to THA after an ipsilateral lower extremity amputation.

One of the earliest reports on THA in amputated patients dates back to 1953, and it was published by Gills et al. [42]. They described six patients with hip osteoarthritis (OA), three of whom had ipsilateral AKA. The patients underwent arthroplasty by metallic implant, and post-operatively, a plaster spica was used on the stump for three weeks, followed by two weeks of exercise before fitting the prosthetic limb. The outcome in all cases was the relief of osteoarthritic pain.

In 2010, Sathappan et al. [25] presented a comprehensive case report of THA implant in one AKA 40-year-old patient with post-traumatic arthritis, who had sustained bilateral pelvic fractures, a right acetabular fracture, and AKA after a war injury. Surgery was performed by a standard posterolateral approach; the dislocation was not possible because of the significant posterior heterotopic ossifications, and the patient required a trochanteric osteotomy followed by in situ femoral neck osteotomy and capsular release. The greater trochanter was reconstructed after THA implant using a trochanteric cable grip system. THA resulted in an overall improvement of hip flexion, abduction, and rotation at the reported 10-month follow-up. The authors emphasized the importance of the pre-op study to determine the patient’s residual limb length, which can predict rehabilitation potential. They also stressed the need for implant retroversion avoidance by using Ranawats’ test for rotation and Shuck’s test for fixation to verify the correct position of the components to reduce the risk of dislocation.

Leonard and Nicholson [28], in 2010, published the case report of a young woman with arthrogryposis, predominantly affecting the right lower limb. She underwent several operations as a child and young adult, resulting in an AKA and fitting with an exo-prothesis; she had fixed flexion deformity of 15°, flexion to 70°, and virtually no rotation. They performed THA through a anterolateral approach; they used a 5 mm Steinman pin on a large t-handle to the distal femur and a bone clamp to control the rotation and to provide sufficient and safe torque to allow the broaching of the femoral canal. At a 5-year follow-up, they reported no pain and good ambulation with a prothesis.

Diamond et al. [22] operated on a 43-year-old female that underwent THA 18 years after AKA. The patient had a short femoral stump, 130 mm long from the tip of the greater trochanter to the distal end of the shaft, that required the use of a short hip stem implant. To control hip dislocation, a Steinman pin was applied at the great trochanter. The patient showed clinical improvement and increased hip functional scores at the 2-year follow-up, using an above-knee prosthesis for weight bearing.

Ma et al. [23] reported a 67-year-old male with a history of right post-traumatic AKA, who later suffered an acute femoral neck fracture. A total hip replacement was completed, using a Steinmann pin placed at the greater trochanter to help with dislocation. The results were good, with improvement in hip joint activities, no pain, and unrestricted walking with artificial limb after two years. Steinmann pin placement at the greater trochanter was considered easier and decreased infection risk, contrary to placing it at the distal femur. Longer and more intense rehabilitation protocols are usually required compared with a standard THA.

Malagelada et al. [31] described one of the first cases of arthroplasty in AKA patients with chronic vascular disease at both lower limbs. The hip was approached via a standard posterolateral approach. At 18 days after surgery, the patient underwent surgical debridement and implant retention with revision of the modular components for infection. When dealing with patients with comorbidities, the associated increased rate of complications should be considered; moreover, the use of cemented implants should be taken into consideration because of decreased bone mineralization and widening of the medullary canal in those fragile patients.

Patnaik et al. [34] recently reported a young male who underwent an AKA for a severe road accident, resulting in right femur fracture with vascular compromise and right hip dislocation that required open reduction. Two months after, the patient developed post-traumatic OA with head avascular necrosis and deformity, requiring a THA procedure. Surgery was performed via an anterolateral approach, supporting the choice with the aim to reduce the risk of dislocation while wearing an exo-prothesis [39]. The authors used a bone clamp to intraoperatively manage the residual limb.

Triphaty et al. [32], reported the case of a young male who suffered from a serious accident that left his right limb insensate, and that subsequently developed avascular necrosis of the femoral head. The authors performed an AKA and THA during the same surgery, the first one with a supine approach and the second one in left lateral decubitus using a posterior approach. They used a lateral Steinmann pin placed at the distal part of the femur through the surgical wound of the AKA to help them dislocate the hip. The authors reconsidered the choice to perform both surgeries at the same time, but emphasized the role of stump exercise, which helped the patient to recover from the previous muscle atrophy to use an external prothesis.

The most recent review was published in 2023 by Galloway et al. [33].; they reported on 21 THAs and 23 total knee artrhoplasty (TKA) procedures performed on 39 patients with lower extremity amputation between 2002 and 2022. Six of the THA cases were ipsilateral to the amputated limb, and most components were uncemented. Even if this study presents heterogenous data, with not a clear specification between AKA and BKA on ipsilateral amputated patients, it is included in our review because it provides interesting and up-to-date data. Within the THA group, the 10-year revision rates were 4.8% and the average Oxford Hip Score (OHS) post-operative was 40 for ipsilateral cases and 41 for contralateral cases, and the average EQ5D Visual Analogue Scale result was 59.1 for the THA cohort. Despite being very heterogeneous, this is probably the largest study utilizing these PROMS for the assessment of lower extremity amputees receiving total arthroplasty surgery, showing the effectiveness of this procedure in these patients.

## 2. Discussion

THA performance in the amputee, either AKA or BKA, requires a close evaluation and pre-operative planning to reduce the risk of perioperative complications and to optimize the outcomes. Most patients requiring THA are post-traumatic amputees, and require evaluation of periarticular heterotopic ossifications, skin and soft tissue conditions at the stump level, and presence of associated bone injuries, including fractures of the pelvis and non-unions. These associated lesions and ancillary medical conditions, if correctly identified and studied pre-operatively, may require some invasive procedures during THA surgery, including a trochanteric osteotomy, and soft tissue management [25]. Vascular and diabetic amputees may otherwise not be suitable for the procedure in most cases, and these patients usually undergo THA primarily in the case of femoral neck fractures; however, these patients are specifically exposed to an increased risk of infection and wound problems that could threaten patients’ life and overall health status; moreover, they might not be suitable to ambulate post-operatively with a wearable prosthesis.

To perform a correct templating, especially in AKA patients, it is important that the radiographs include the entire length of the remaining limb [31,32] to assess the correct femoral anatomy, since it can predict the potential [43] of rehabilitation; sometimes, the residual limb could be very short [22], requiring the use of short implants, and making it more difficult to perform a safe dislocation of the femoral head and stem implant.

Accurate templating with a sufficient estimate of the post-operative limb length is an important part of the pre-operative planning; an incorrect position of the stem could lead to altered axis of the femur with pathological offset, determining an incorrect or even painful use of an external prosthesis [38]. In fact, the correct angle between the neck and the shaft is probably the most important relationship to recreate in an amputated patient to restore the function of hip abductors [35]. Assessment of bone quality and density is required pre-operatively, because it can be poor at the amputated ipsilateral limb [10,18]. If present, it is related to an increased risk of intraoperative fractures, which may hamper the rehabilitation process, and may require expansion of the surgical field; in those patients, the use of cemented implants should be considered. The decreased cortical area is common at the end of the stump in amputated patients [10], and it could determine a widening of the medullary canal; for this reason, some authors advice for the default use of a cemented THA implants [26,31] to decrease the risk of intraoperative fractures and to promote immediate weight bearing.

**Figure 2:**
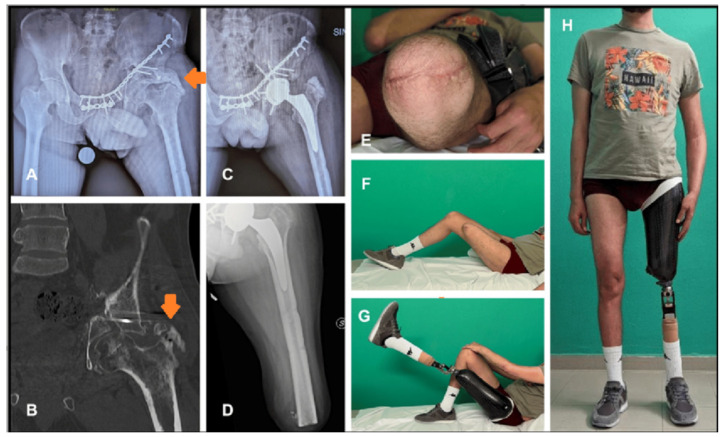
A case of total hip arthroplasty(THA) in a post-traumatic above-knee-amputated (AKA) patient with pelvic non-union after the fracture was managed by fixation with plate and screws; severe secondary hip arthritis developed, together with massive periarticular heterotopic ossification (orange arrows). (**A**,**B**). Ossification removal and total hip arthroplasty were performed via a posterolateral approach, using two Steinman pins to intraoperatively dislocate the femoral head (**C**). Revision of the stump was required to improve soft tissue coverage, approximately 2 months after THA, and it healed uneventfully (**D**,**E**). At 6 months follow-up, patient had fully regained hip range of motion and recovered the iliopsoas muscle (**F**,**G**), and ambulated with an external prothesis and a cane (**H**).

Safe exposure and dislocation of the proximal femur is a major issue in the amputated patient undergoing THA, and the surgeon should carefully plan this step in advance to optimize the outcomes of the surgery and to limit the risk of complications and intraoperative fractures. Many patients have associated periarticular calcifications that may hamper the exposure of the hip joint (Figure 2). Moreover, especially in AKA patients, the short lever of the residual limb and the absence of the knee articulation could increase the difficulty of surgery. The use of a Steinmann pin placed at the femur shaft or at the trochanteric area, used as a lever to apply the correct force for femoral head dislocation, is commonly performed in this patient population [20,21,26,30]. Pins can be placed on different parts of the femur according to the surgeons’ preference, leaving some distance from the end of stump if possible; however, some authors advocate against their use [32] because of the risk of intraoperative fractures in the case of osteopenic areas. This feared complication might occur when elevated forces are applied during the dislocation maneuver.

**Figure 3:**
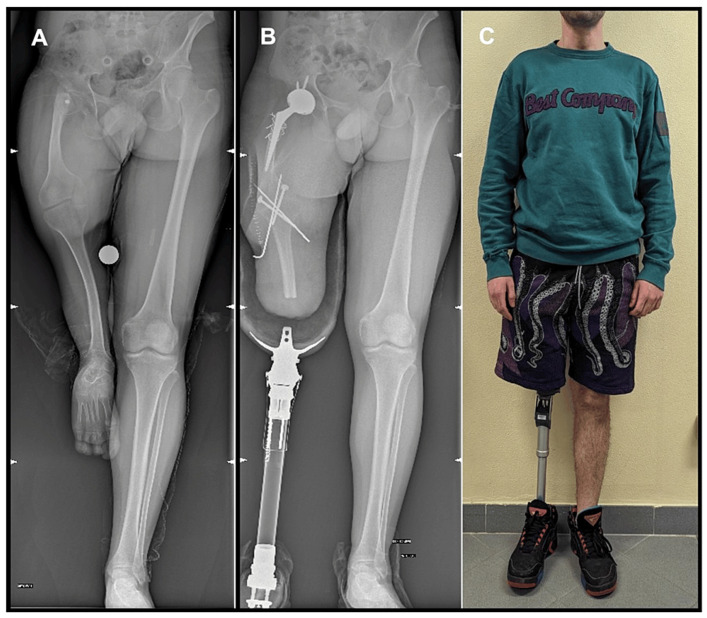
Patient with hypoplasia of the right limb (**A**) who underwent a total hip arthroplasty (THA) on the ipsilateral side, after a below-knee amputation with knee arthrodesis. Patient used a prosthesis early after surgery through a cast to condition the limb to its use, allowing the patient to wear and ambulate with an external prothesis as an above-knee-amputated patient (**B**). At 12 months follow-up after THA, the patient walks with an external prothesis and no aids (**C**).

Surgical planning also requires the evaluation of the ability of operated patients to restore gait and to wear a prosthesis effectively. Rehabilitation programs should be clearly set, keeping in mind the anatomy of the amputated lower limb, considering which muscles are mostly affected by the level of amputation, such as being detached in some cases, or weak for prolonged disuse in others. Since the gastrocnemius is the main propulsive muscle during walking, its absence in the amputee patient requires an increased activity of the residual muscles. Hip extensors, gluteal muscles, and hamstrings are protected from atrophy by their new role. In contrast, due to the decrease in knee flexion during loading, the quadriceps reduces its work, and its fibers tend to become hypotrophic with the exception of the component of the rectus femoris muscle [44]. During stance on the ipsilateral side, the hip flexors, extensors, and external rotators become the main power generators, while the abductors and adductors become the main absorbers, in stark contrast to the contralateral side [45]. Therefore, in addition to a typical THA post-operative rehabilitation regimen, hip flexor and knee extensor stretching are frequently deemed important exercises [23,26]. Aggressive physical therapy may be started pre-operatively targeting these muscles, leading to an early recovery of gait ability, faster recovery, and shorter hospital stay [25,38]. BKA patients often have higher functional demands, necessitating a challenging post-operative rehabilitation program to maximize the outcomes. Although some patients still use a cane for walking after three months, many relatively young patients do not require any aid to support ambulation after THA (Figure 3). BKA patients often start ambulating with their prosthesis and an aid within the first 14 days after THA; for this reason, the promotion of an expedited ambulation protocol may mitigate post-operative limb swelling. The predominant etiology in AKA cases is post-traumatic osteoarthritis, with occasional instances of post-vascular amputation, arthrogryposis, and fractures. Despite the diverse etiologies, all AKA patients eventually achieve independent ambulation with a prosthesis, although sometimes it requires more time compared to BKA patients, ranging from 3 months to 2.5 years [22,25,28,31]. AKA patients require a careful and balanced physical therapy to achieve a satisfactory gait mechanism because of the significant loss of muscle mass and decline in adduction function, usually associated with muscle contractures [25,34].

## 3. Conclusions

THA in amputated patients requires a comprehensive and careful approach, given the multitude of challenges that these present when surgery is required. An extensive pre-operative workup considering the patient’s history, comorbidities, and reasons for amputation is essential.

Our narrative review has limitations. First, we acknowledge the nature of a narrative review itself, which cannot have the more precise revision criteria that a systematic review offers. However, we consider this a more appropriate study design due to the small and heterogeneous group of studies, which include a large case mix, implant manufacturers, and amputation types. Some of the study did not include extensive information about post-operative therapy, or long-term follow-up data.

Recognizing that each patient, based on the level of amputation, has a specific gait type and distinct functional requirements is the key to a successful surgery. It is suggested to be mindful of the anatomical differences and the bone changes in the femur requiring THA, sometimes through cemented stems, and to select the most effective and safe surgical approach among those available to surgeons. Muscle and soft tissue reconstruction promote an early and effective rehabilitation program to achieve early weight bearing and ambulation recovery wearing an external prosthesis.

## Figures and Tables

**Table 1:** Clinical studies of total hip arthroplasty (THA) on ipsilateral below-knee amputated (BKA) patients. Total hip arthroplasty (THA), below-knee amputated (BKA), through-knee amputated (TKA), above-knee amputated (AKA), standard deviation (SD).

Authors	Study	Patients with THA on Ipsilateral Amputated Limb (23)	Etiology of Amputation	Times from Amputation to THA	Reason for THA	Surgical Data	Physical Therapy	Follow-Up
Nancy M. Prickett et al. [38]	Case report	1 BKA	Osteomyelitis	17 ys	Fracture	Posterior approach	Pre-op isometric contractions of the quadriceps and gluteal. Day 3, ambulate with walker. Final ROM: flexion 105°, abduction 30°, and extension 0.	8 weeks: walk with exo-prosthesis.
								9 weeks: ambulate w/o aids.
Edward J. Nejat et al. [39]	Retrospective series	2 BKA	Digenesis, osteomyelitis	10 ys, 0.058 ys	AVN femur head, secondary OA	No data	Patient 1: Day 1, ambulate walker, Day 5, ambulate with crutches.	Patient 1: at last follow-up, walks with no aids. Patient 2: 3 months ambulate with walker and prothesis.
							Patient 2: Day 3, stands with walker.	
							No final ROM data.	
Moshe Salai et al. [29]	Retrospective series	4 BKA	Post-traumatic, peripherical vascular disease, diabetes	2.58 ys, 2.16 ys, 1.58 ys, 3.58 ys	Fracture	Cementless components, anterolateral approach	No physical therapy data.	Mean follow-up, 5.5 ys. Two patients ambulate with no aids, two required a cane at last follow-up.
Jeson Mak et al. [40]	Case report	1 BKA	Peripheral vascular disease	30 ys	Secondary OA	Posterior approach, large bearing metal-on-metal prosthesis	Day 5, started gait retraining.	30 days: gait with prothesis and cane.
							Day 8, first use of prothesis.	
							Final ROM: internal rotation 5°, external rotation 10°, abduction 35°, adduction 10°, flexion 95°, and extension 20°	
Karim Masmoudi et al. [26]	Case report	1 BKA	Post-traumatic ischemia	54 ys	Fracture	Cemented metallic component, lateral approach	Day 3, ambulate with prosthesis and crutches.	3 years: walk with one cane.
							Physical therapy included isometric strengthening of the hip abductors and flexors.	
							Final ROM flexion 90°, external and internal rotation 30°, abduction 40° and adduction 20°.	
Derek F. Amanatullah et al. [41]	Retrospective series	18: 14 BKA 2 TKA 2 AKA	Vascular, diabetes, osteomielites, trauma, tumor, neuropathy	Not specified	Not specified	Various surgical approaches, mostly anterior	No physical therapy data.	Average follow-up, 4.9 years. No difference between groups for failure.

**Table 2:** Clinical studies of total hip arthroplasty on ipsilateral above-knee amputated patients. Total hip arthroplasty (THA), below-knee amputated (BKA), through-knee amputated (TKA), above-knee amputated (AKA), standard deviation (SD), years (ys).

Authors	Study	Patients with THA on Ipsilateral Amputated Limb (14/2)	Etiology for Amputation	Times from Amputation to THA	Reason for THA	Surgical Data	Physical Therapy	Follow-Up
Sathappan S. Sathappan et al. [25]	Case report	1 AKA	Trauma	11 ys	Secondary OA	Posterolateral approach.	Physical therapy pre- and post-op with active assisted ROM exercise.	10 Months: walk with prosthesis
						Uncemented components.	Isometric hip abductor strengthening.	
						Trochanteric osteotomy for surgical dislocation.	Final ROM: flexion 85°, abduction 40°, internal and external rotation 30°.	
Francesc Malagelada et al. [31]	Case report	1 AKA	Peripheral vascular disease	2 ys	Secondary OA	Cemented components, posterolateral approach.	Day 5, mobilize with prothesis and aid.	Day 18: surgical washout and revision for infection
						Use of bone clamp to dislocate femur head.	No final ROM data.	3 months: ambulate with prothesis
Michael Leonard et al. [28]	Case report	1 AKA	Infection	/	Secondary OA	Uncemented components, antero-lateral approach.	No physical therapy data.	5 years: Ambulate with prosthesis
						Use of a Steinman pin and a bone clamp to control the stump.		
Owen J. Diamond et al. [22]	Case report	1 AKA	Trauma	18 ys	Secondary OA	Uncemented components with short stem, posterior approach.	Week 8, full weight bearing, ambulate with crutches.	2 Years: Ambulate with crutches
						Use of a pin at the greater trochanter.	No final ROM data.	
Leon Gillis et al. [42]	Case series	3 AKA	Trauma	34 ys, 5 ys, 34 ys	Secondary OA	Cemented metallic components.	No physical therapy data.	Patient 1: ambulate with crutches
								Patient 2: ambulate with artificial limb
								Patient 3: ambulate with a pylon.
Chunhui Ma et al. [23]	Case report	1 AKA	Trauma	47 ys	Fracture	Posterior approach.	Week 3, physical therapy wearing artificial limb, gait retraining.	2 Years: ambulate with artificial limb without pain.
						Use of Steinmann pin at the greater trochanter.	Intense physical therapy targeting hip abductors.	
							No final ROM data.	
Murat Pekmezci et al. [24]	Case report	1 TKA	Trauma	45 ys	Secondary OA	Posterolateral approach.	Week 8, return to full-time prosthesis use.	18 months: ambulate with prothesis and no aids.
						Use of bone clamp and Steinman pin at the distal femur.	No final ROM data.	
Hassan Boussakri et al. [27]	Case report	1 AKA	Peripheral vascular disease	11 ys	Fracture	Lateral approach.	No physical therapy data.	2.5 years: ambulate with prosthesis and no pain.
						Use of a bone clamp.		
Sanjeev Patnaik et al. [34]	Case report	1 AKA	Trauma	0.25 ys	Secondary OA	Uncemented components, anterolateral approach.	Day 2, ambulate with prosthesis on full weight bearing.	12 months: ambulate with no aids.
						Use of bone clamp in lesser trochanteric area.	No final ROM data.	
Sujit Tripathy et al. [32]	Case report	1 AKA	Trauma	Same time	Vascular damage	AKA and THA during same surgery session, first supine and second lateral approach, uncemented components.	Day 2, isometric exercises.	4 months: ambulate with final prosthesis
						Use of Steinmann pin at the end of femur shaft.	Full weight bearing with previous prosthesis and walking aid.	
							No final ROM data.	
Derek F. Amanatullah et al. [41]	Retrospective series	18: 14 BKA 2 TKA 2 AKA	Vascular, Diabetes, osteomielites, trauma, tumor, neuropathy	Not specified	Not specified	Several surgical approaches, mostly anterior.	No physical therapy data.	Average follow-up, 4.9 years. No difference between groups for failure.

## Data Availability

Data sharing is not applicable. No new data were created or analyzed in this study.
